# “You’ll Be Chased Away”: Sources, Experiences, and Effects of Violence and Stigma among Gay and Bisexual Men in Kenya

**DOI:** 10.3390/ijerph20042825

**Published:** 2023-02-05

**Authors:** Katherine A. Lewis, Laura Jadwin-Cakmak, Jeffrey Walimbwa, Adedotun Ogunbajo, Juan C. Jauregui, Daniel Peter Onyango, Darius M. Moore, Gabriel Lee Johnson, Wilson Odero, Gary W. Harper

**Affiliations:** 1 Department of Community Health Sciences, Fielding School of Public Health, University of California Los Angeles, Los Angeles, CA 90095, USA; 2 Department of Health Behavior and Health Education, University of Michigan School of Public Health, Ann Arbor, MI 48108, USA; 3 Ishtar MSM, Nairobi 00100, Kenya; 4 RAND Corporation, Santa Monica, CA 90401, USA; 5 Luskin School of Public Affairs, University of California Los Angeles, Los Angeles, CA 90095, USA; 6 Let Good Be Told In Us, Nyanza, Rift Valley, and Western Kenya (NYARWEK) LGBTI Coalition, Kisumu 40100, Kenya; 7 School of Medicine and School of Public Health, Maseno University, Kisumu 40100, Kenya

**Keywords:** gay and bisexual men, Kenya, stigma, violence, qualitative research

## Abstract

Gay and bisexual men in Kenya face extreme socio-political stigma which manifests in widespread violence and discrimination across socio-ecological levels. We conducted individual in-depth interviews with 60 gay and bisexual men in western and central Kenya. Interview transcripts were thematically analyzed using an inductive, phenomenological approach to qualitatively examine experiences of stigma and violence at the interpersonal and institutional levels. A total of seven primary themes and four sub-themes emerged from the data. At the interpersonal level, participants described stigma and violence from family, friends, and romantic/sexual partners with sub-themes for gay-baiting violence, blackmail, intimate partner violence, and commitment phobia. At the institutional level, participants described stigma and violence from religious, employment, educational, and healthcare institutions. This stigma and violence severely impacted the lives of participants including their mental health, physical health, sexual health, socioeconomic status, and ability to access health-promoting services. These data identify sources of stigma and describe how this stigma manifests in the everyday lives of gay and bisexual men in Kenya. Study findings and quotes from participants highlight the severity of violence, stigma, and discrimination faced by this community and emphasize the need for decriminalization of same-sex sexualities as well as interventions to support health and wellbeing.

## 1. Introduction

Gay and bisexual men in Kenya face pervasive identity-based stigma and violence across socio-ecological levels [1,2,3,4]. Despite the commitment to human rights in Kenya’s constitution and growing calls by human rights organizations for an end to violence against lesbian, gay, bisexual, transgender, and queer (LGBTQ+) communities, colonial-era laws prohibiting same-sex sexual behavior have persisted [5,6,7,8,9]. Under sections 162 and 165 of the Kenyan Penal Code, consensual same-sex sexual behavior is criminalized and punishable by up to 14 years in prison [10]. This is compounded by dominant cultural and religious beliefs strongly rooted in heteronormativity and sentiments opposed to LGBTQ+ identities [11,12]. Heteronormativity, strictly established gender roles, and norms surrounding masculinity, negatively impact the health of gay and bisexual men and remain extremely persistent, even given the presence of accepted forms of same-sex unions in pre-colonial Kenya [2,11,12,13]. For example, in one study of Kenyan religious leaders, 95% agreed with anti-LGBTQ+ sentiments and 32% endorsed the use of violence to preserve cultural values [14]. Fueled by this atmosphere of socio-political marginalization, human rights violations against the Kenyan LGBTQ+ community persist.

In the face of the growing visibility of Kenyan LGBTQ+ activism, criminalization of same-sex behavior and extreme cultural stigma continue to facilitate harassment, discrimination, and violence [1]. In a study of LGBTQ+ adults in Kenya, Harper et al. found that 38% of gay and bisexual men and other men who have sex with men (GBMSM) reported ever experiencing sexual minority-based violence [3]. In line with these findings of high rates of violence and abuse, Secor et al. found that 47% of GBMSM in Mtwapa, Kenya reported at least one experience of forced sexual intercourse or upsetting sexual experience with a related adult or authority figure before the age of 17 [15]. Another study of GBMSM in Kisumu, Kenya found that 81% of participants reported any childhood physical or sexual abuse [16]. These studies highlight the urgent need for data to better understand how violence and stigma impact the lives and health of gay and bisexual men in Kenya.

Reports from the Human Rights Watch (HRW) and the Kenya Human Rights Commission (KHRC) have presented qualitative data highlighting the severity of violence, harassment, and discrimination faced by Kenya’s LGBTQ+ community [1,2]. In their report, The Issue is Violence, HRW provides qualitative data from community members describing brutal and highly prevalent physical and sexual violence perpetrated by state actors and civilians [1]. Similarly, the 2011 KHRC report, The Outlawed Amongst Us, describes persistent violence and stigma faced by the Kenyan LGBTQ+ community including blackmail, physical violence, sexual violence, medical research abuse, expulsion from schools, rejection from family and friends, and harassment from healthcare providers and religious groups [2]. Both of these reports noted that the criminalization of same-sex sexual behavior coupled with pervasive cultural and religious anti-LGBTQ+ sentiment fueled this violence, dissuaded victims from reporting incidents, and prevented perpetrators from being caught [1,2].

### 1.1. Stigma, Minority Stress, and Health Outcomes

Link and Phelan’s widely used conceptualization of stigma describes overlapping components by which groups of people are distinguished, labeled, and experience status loss based on dominant cultural beliefs that distinguish desirable and undesirable characteristics [17]. This conceptualization includes separation, negative stereotyping, and discrimination based on characteristics that are deemed undesirable [17]. Anti-LGBTQ+ stigma negatively impacts health outcomes and occurs across socio-ecological levels [18]. This includes internalized stigma (intrapersonal level), assault and rejection (interpersonal level), and discriminatory policies (structural level) [18]. Meyer’s Minority Stress Model describes how violence and stigma have downstream implications for health outcomes [19]. This model characterizes experiences of discrimination and violence as distal minority stress processes, which, coupled with proximal minority stressors (e.g., concealment and internalized stigma) and general life stressors (e.g., financial stress), impact mental health outcomes [19]. This relationship between stressors and mental health outcomes is buffered by coping and social support, which are described by Fergus and Zimmerman as resilience-focused assets and resources [20]. Although the Minority Stress Model originated in the US, it has proven to be a useful tool for studying the health of gay and bisexual men in Kenya [3,4] as well as other Sub-Saharan African countries [21,22,23,24,25,26]. Similarly, resilience frameworks have been used in studies of gay and bisexual men in Kenya to highlight the strengths of this community in the face of adversity and to create a research paradigm shift towards strengths-based rather than deficit-based research [27].

Despite the recognition of the growing burden of mental health concerns across Kenya and Sub-Saharan Africa, limited data exists to examine the mental health of Kenyan gay and bisexual men, as most studies focusing on this population are focused on sexual health and HIV prevention/treatment [3,27,28,29]. However, the available data, as predicted by the Minority Stress Model, reveal a high burden of unaddressed mental health challenges. For example, Harper et al. found that 12% of GBMSM in Kenya reported clinically significant levels of psychological distress, 52% reported clinically significant PTSD symptoms, and 28% reported clinically significant depressive symptoms [3]. Similarly, Kunzweiler et al. [16] found that 11% of GBMSM participants in Kisumu, Kenya reported severe depressive symptoms and 50% reported harmful alcohol abuse, while Secor et al. found that 38% of GBMSM participants reported ideations of suicide or self-harm more than half of the days in the past 2 weeks [15]. In contrast, national rates of mental health concerns among Kenyans are much lower with prevalence estimates at 12.6% for depression, 15.7% for anxiety, and 4.5% for PTSD [29]. These alarming rates of mental health concerns for gay and bisexual men not only demonstrate the impact of extreme stigma on health outcomes, but also highlight the need to better understand how stigma and violence impact the lives of gay and bisexual men in order to create effective interventions for mental health promotion.

### 1.2. Socio-Ecological and Qualitative Approach

The socio-ecological model is a useful tool for the conceptualization of the multi-faceted stigma faced by Kenyan gay and bisexual men, as well as the resilience assets and resources which may serve as a buffer between this stigma and associated negative health outcomes [18,30,31,32]. Examining elements of stigma or resilience at different socio-ecological levels not only provides a deeper understanding of the phenomenon, but also may facilitate the creation of interventions which target specific levels to improve health outcomes [27,33]. For example, Harper et al. [30] found that perceived social support was associated with condom use and higher self-esteem among GBMSM in western Kenya. Additionally, Graham et al. [34] identified stigma in healthcare facilities and unsupportive family, friends, and healthcare providers as significant barriers to wellness among Kenyan gay and bisexual men living with HIV. By identifying interpersonal and institutional sources of violence and stigma, as well as describing how this stigma manifests in the lives of Kenyan gay and bisexual men, this paper will expand upon the limited existing literature and will highlight areas for future intervention.

In addition to a socio-ecological approach, a qualitative phenomenological approach is used to focus directly on the voices and experiences of community members. Qualitative approaches have been used in other studies and reports on the health of Kenyan GBMSM and have highlighted the impact of topics such as access to affirming healthcare [34], intrapersonal resilience assets [27], social and familial support [33,34], and interpersonal violence [1,2]. This paper is unique in that the combined socio-ecological, qualitative, and phenomenological approaches will identify areas for intervention at the interpersonal and institutional levels by identifying sources of stigma and describing the manifestations and effects of this stigma in the lives of community members.

## 2. Materials and Methods

We conducted qualitative individual in-depth interviews (IDIs) with 60 gay and bisexual men in western and central Kenya between the ages of 18 and 50, using a two-level stratified purposive sampling strategy. Our first level of stratification was geographic location (Kisumu in western Kenya and Nairobi in central Kenya), and our second level was age range (18–24 years; 25–34 years; 35–50 years). We sought for diversity of sexual orientation identities (e.g., gay, bisexual) within each stratification level. Inclusion criteria included: (a) assigned male sex at birth and currently identifies as a man; (b) self-identifies as gay, bisexual, or another non-heterosexual identity; (c) ages 18–50 inclusive; (d) ability to speak English or Kiswahili; and (e) willing and able to provide informed consent and participate in an IDI. Overall, we sought to recruit individuals who were perceived to be good key informants, defined as a person who thinks about the study topics, is comfortable talking about these topics, and is good at describing their thoughts and feelings.

Research team members, the majority of whom were Kenyan gay and bisexual men, worked together to create an outline for topics to be discussed in the interview guide. Through a series of in-person meetings in Kenya, the team created the initial interview guide and then worked together to reduce the number of questions and refine the wording to be concise and clear. Throughout the course of qualitative interviewer training, modifications were made to the guide to assure its utility with gay and bisexual men in both Kisumu and Nairobi. The interview guide was grounded in phenomenological and constructivist frameworks, which provided a general structure for discussion but required participants to provide their own conceptualizations of terms and phrases based on their lived experiences [35,36]. The guide included a series of questions and probes focused on 15 primary areas: masculinity, revealing sexual identity to others, sexual positioning, relationships, gay culture and connections with other gay men, older gay men, resilience, sexual and reproductive health and rights, alcohol and substance use, mental health, education and employment, religion and spirituality, violence, family of origin, and hopes and dreams for the future. The structure and content of the questions did not follow any a priori theory or framework; thus, we were able to conduct an inductive inquiry of participants’ thoughts, feelings, and experiences in these general areas as described by the participants.

All study activities were conducted by members of the research team who are gay or bisexual Kenyan men who work directly with LGBTQ+-specific community-based organizations (CBOs) and clinics. A total of five interviewers conducted these interviews across our two sites—Kisumu and Nairobi. Interviews were conducted from July to September 2018. Given the purposive nature of our sampling frame, we recruited participants through outreach activities conducted by our interviewers at CBOs and health clinics in Kisumu and Nairobi that provide services to gay and bisexual men. Recruitment and screening took place verbally with men who fit criteria in accordance with our inclusion criteria and our stratified sampling framework. All interviewers followed safety protocols, and no specific recruitment materials were used in order to provide a greater degree of safety and security for interviewers and potential participants.

Interviews took place in private rooms at one of our CBO or clinic research sites. Interviewers first obtained verbal consent for research participation, then verbally administered a brief demographic survey. They then conducted the IDI and recorded it using a digital audio recorder. Following the interview, the interviewer debriefed with the participant and then provided him with a monetary incentive and a resource guide that provided information on an array of physical and mental health services that are friendly to gay and bisexual men. Interviews lasted 60 min on average, ranging from 30 min to 2 h. Interviews were primarily conducted in English with a mix of some Kiswahili, based on the most comfortable language for the participant. All interviewers were fluent in both languages. Recordings were transcribed by other research team members, and then all transcripts were de-identified and quality checked by team members to ensure accuracy of transcription. The Institutional Review Boards of the participating US academic institutions, as well as the Ethics Review Committee of our local Kenyan academic partner approved all study procedures.

In order to ensure the credibility of our findings and rigor of our qualitative methods, we incorporated prolonged community engagement, persistent observation, triangulation, and member checking as strategies for this analysis [37,38]. Senior members of this team have worked with LGBTQ+ communities in Kisumu, Kenya for over twelve years on both research and community-building efforts including social/cultural events and community organizing. Triangulation techniques included use of five different interviewers and six analysts from varying fields including public health, psychology, and social work. Members of the analytic team represented diverse racial/ethnic identities, sexual orientations, gender identities, and educational attainment. Since the analytic team was not comprised of gay and bisexual Kenyan men, all results were presented to the team of interviewers (all gay and bisexual men from Kenya) who provided additional insights into the findings as a form of member checking [37].

To initiate the coding process, analysts were trained in the study methods and procedures as well as cultural contexts for gay and bisexual men in Kenya and qualitative data analysis techniques grounded in a phenomenological inquiry framework [35,36]. Phenomenology is specifically focused on describing what a given group of people has in common as they experience the same or similar experiences or phenomena. It is an inductive analytic approach that allows patterns, themes, and categories of analysis to emerge from participant narratives [35,36]. In addition, phenomenology is rooted in a social constructionist standpoint; thus, individual and collective lived experiences are constructed as reality within the phenomenological framework. In line with this approach, conceptual ‘outliers’ are not silenced but rather presented in the findings along with themes discussed by a larger majority of participants to ensure that all voices are included [35,39]. This analytic approach has been utilized in other studies focused on the lived experiences of gay and bisexual men in Kenya [27,33].

An inductive consensus-building process was used to develop the original research question (What factors negatively affect the mental health of gay and bisexual men in Kenya?). After team members had each read five transcripts and brainstormed common themes, transcripts were assigned to all analysts, ensuring variability and overlap such that each transcript was read by two team members. Coders engaged in open coding which involved noting key illustrative quotes and applying codes to the transcripts. The analysis team met weekly to develop a formal codebook and operationally define all included codes. As coding progressed, the team also made modifications to the codebook as needed which included adding, collapsing, eliminating, and splitting codes. Due to the depth of findings and the wide breadth of the original research question, the results were split into several different manuscripts. The current manuscript focuses on gay and bisexual men’s experiences of violence and stigma at interpersonal and institutional levels.

Demographics of our sample are provided in Table 1. Participants ranged in age from 20 to 46 (mean = 29.3) and all identified as gay/homosexual (76.3%) or bisexual (23.7%). Most participants reported that their highest educational level was a diploma (40.7%) or secondary school (25.4%), and most either worked full-time (42.3%) or part-time (39.0%). All participants resided in Kisumu or Nairobi counties (53.3% and 46.7%, respectively), and a majority identified as Luo (55.2%), followed by Kikuyu (13.8%) and Luhya (12.1%). Additionally, 96.7% of participants reported a religious affiliation, with Catholic (37.3%) and Anglican (23.7%) being the most common.

## 3. Results

Our analyses identified manifestations of stigma and violence within both intimate interpersonal relationships and critical social institutions. Within the intimate interpersonal relationships domain, there were three primary thematic areas in which stigma and violence were described by participants, including family, friends, and romantic and/or sexual partners. Sub-themes of gay-baiting violence, blackmail, intimate partner violence, and commitment phobia were further identified within the romantic and/or sexual partners primary theme. Within the critical social institutions, there were four primary thematic areas in which participants reported experiencing stigma and violence, including religious, employment, educational, and healthcare institutions. The final thematic code tree is illustrated in Table 2. Detailed descriptions of the ways in which participants experienced stigma and violence within each of these domains, as well as the impact on their well-being, are provided with supporting quotes, along with a participant pseudonym and relevant demographic information (age, sexual orientation identity, and geographic location). Of note, the themes identified were discussed by more participants than just those quoted, but these illustrative quotes have been chosen for their representativeness of the larger dataset.

### 3.1. Intimate Interpersonal Relationships

Within intimate interpersonal relationships, three primary themes emerged with four sub-themes for one of the primary themes. Participants described violence and stigma from family, friends, and romantic and/or sexual partners, with extended information regarding gay-baiting violence, blackmail, intimate partner violence, and commitment phobia when discussing violence from romantic/sexual partners.

#### 3.1.1. Family

Manifestations of stigma and violence from family members included harassment, rejection, and/or physical violence. Although some participants described positive relationships with family members, experiences of stigma, violence, and rejection were frequently discussed in the context of being forced or pressured into heterosexual marriage, being beaten, being kicked out of the parents’ house, being financially cut off, or being told that they were not a “real” member of the family. Coming out to one’s family as gay or bisexual was described as a careful balance of anticipating whether one’s parents would be accepting or rejecting. There was also a financial aspect that sometimes influenced parents’ responses, in that one’s parents would be more accepting of their son’s sexuality if he was providing financial support to the family. Below, Davy (24, gay, Kisumu) describes the consequences of being rejected from family.

*“…You’ll be chased away…it means that they have denied you the basic…items that you need from them. This includes clothing, housing, and food. And from there you have to fend for yourself…that’s why there’s also been a rising cases of HIV because on the streets, somebody will come to you and wants condom-less sex, and ‘cause you’re desperate for that money…you give in.”*

Family rejection was described as having severe mental and physical health consequences including alcohol use/abuse, depression, suicide, and risky sexual practices for emotional or financial security. Below, Joseph (31, gay, Nairobi) describes the mental health challenges associated with being rejected by family.

*“Because you have rejections, stigma…there’s stress, there’s depression…Some families will kick you out of their house or their lives. Some will even beat you. As a result…you might want to attempt suicide at times…”*

#### 3.1.2. Friends

Manifestations of stigma and violence from friends included harassment and rejection, primarily discussed in the context of coming out and the potential danger of losing friends as a result. Participants who had not come out to their friends described a fear that their friends would reject them if they did so, and thus, they described concealment as a better option. Below, John (age unknown, gay, Nairobi) describes a personal account of rejection.

*“…Some of them [friends], they will not understand…I had one and once I told him that I am gay, he said ‘Now I don’t want anything to do with you.’…you feel bad. You lost a friend, maybe you are, a good friend, and you lost him.”*

Many participants discussed losing friends after coming out and the potential betrayal and gossip that followed, resulting in them having to make new friends who were more supportive of their sexual orientation. Below, Wafula (24, gay, Kisumu) describes the pain of losing friends after coming out.

*“…you know at times when someone finds out you are gay, let’s say with a friend, it tortures you mentally. …You’ve been friends for let’s say about four years, and then he comes to find out you are gay, and he’s like ‘no I can’t stand you’. And this [is] someone who you’ve been there for and has been there for you, and then out of the blues—actually no not out of the blues. After knowing that you’re gay, he’s just like ‘dude I’m done with you’. That mentally torture.”*

#### 3.1.3. Romantic and/or Sexual Partners

Manifestations of stigma from romantic and/or sexual partners included harassment and violence from current, former, or potential romantic and/or sexual partners, as well as relationship difficulties caused by societal stigma. This includes all types of relationships, including long-term committed relationships, brief sexual encounters (i.e., hookups), and flirting/talking either in person or virtually on dating apps/social media. Participants described gay-baiting violence, blackmail, intimate partner violence, and commitment phobia as manifestations of violence and stigma within these relationships.

##### Gay-Baiting Violence

Gay-baiting violence is a form of violence where the perpetrator pretends to be a gay or bisexual man either physically in person or virtually online (i.e., dating apps or social media) and lures a gay or bisexual man to a secluded location under the illusion of engaging in romantic or sexual activities, then physically attacks him and/or steals his money. With the proliferation of dating apps and social media sites specifically for gay and bisexual men in Kenya, participants noted this as a serious security threat for those seeking romantic or sexual partners online. Erick (24, gay, Kisumu) described how simple attempts at meeting another gay or bisexual man for romantic or sexual connections can result in severe violence.

*“You can meet somebody pretending he is gay maybe you go with him somewhere and he beats you. Or sometimes you can meet somebody on social media. You have a crush in social media and he is straight totally and when you two meet, it’s a fight.”*

##### Blackmail

Blackmail is a form of violence in which a perpetrator forces an individual to give them money/goods or perform sexual acts in exchange for the perpetrator not fulfilling a stated threat—in this case, typically, a threat of disclosing the individual’s sexual orientation to their friends or family. Many participants stated that blackmail has become a significant problem for Kenyan gay and bisexual men, and some described older individuals and those with a higher socioeconomic status as being at particular risk. Participants described experiences in which a sexual partner or ex-partner threatens to tell the participant’s family or friends that the participant had raped the perpetrator, then demands money in exchange for their silence. Below, Ahmed (42, gay, Kisumu) describes blackmail perpetrated by a romantic/sexual partner.

*“… according to me it is love when you meet somebody and love each other, it is not a matter of financial or what. But… after sex he will demand something from you, money or what. So if you don’t give me this much I will shout [tell others about your sexuality]…where I am staying if something like that happens to me, it will be a very big issue…because…I have family there, I have neighbors…”*

##### Intimate Partner Violence

Intimate partner violence refers to physical, emotional, sexual, or other forms of violence perpetrated by a romantic or sexual partner. Below, Douglas (37, gay, Nairobi) describes physical and sexual abuse by a partner.

*“We also have intimate partner, uh, violence, whereby, uh, your partner—you fight with your partner. Like your partner realizes you’re not satisfying him the way he wants, so he forces you to—or he beats you up.”*

Participants described experiencing physical abuse or beatings from their partners, which lead to stress, mental health issues, and negative implications for one’s professional, educational, and social life. Below, Okeyo (27, gay, Kisumu) describes the stigma and fear associated with intimate partner violence.

*“And even within their relationship. People fight. People have been attacked…I went through that one and half years. I never talked about it…It happens a lot. I was almost killed…Yes, there’s stigma…it happens but most cases—it’s not captured because people don’t talk about it.”*

##### Commitment Phobia

In addition to direct experiences of stigma and violence, participants reported that the larger environment of anti-LGBTQ+ stigma impacted their romantic and sexual relationships via commitment phobia, or a perceived difficulty to commit to a long-term romantic relationship. This manifestation of stigma was attributed by some participants to the knowledge that a gay relationship would always have to be concealed (due to criminalization and social stigma), the inability to stay at another man’s house for too long without attracting suspicion, and a lack of a ‘standard’ path of relationship milestones for gay relationships. All of these factors were described as impacting the perceived feasibility of a long-term relationship. Below, Ochieng (24, bisexual, Kisumu) describes the impact of concealing one’s sexual orientation on intimate connections.

*“… some people they will be in a relationship with you but … they want to keep it … discrete…. Maybe the place he stays he doesn’t want people suspecting, questioning…But the person really, he loves you, he is into you but due to the situation around, most people try to- not to keep it that serious relationship.”*

Participants also discussed an inability to be legally married, family pressure to marry a woman, a lack of children, and a lack of role models of ideal gay relationships as well as stereotypes of promiscuity, unreliability, or a preference for hookups over committed, monogamous relationships among gay and bisexual men. Below, Kamau (26, gay, Nairobi) describes the impact of several of these factors on the perceived feasibility of same-sex relationships.

*“We don’t have role models in the gay community who have shown that relationship works. So…getting a serious relationship becomes more hard because of this…Secondly, ‘cause the society in Kenya has made it illegal for people to get married, so people can’t take gay relationships seriously. Lastly is family issues. So, you can’t live with your boyfriend without saying … to a family you’re living with a particular person. So it becomes more hard to have a complete relationship…”*

### 3.2. Critical Social Institutions

At the institutional level, four themes emerged as participants described experiencing stigma and violence within religious, employment, educational, and healthcare institutions.

#### 3.2.1. Religious Institutions

Manifestations of stigma within religious institutions included rejection and harassment from religious leaders and members of the congregation. Although some participants discussed the positive influence of individual-level intrapersonal religious/spiritual beliefs, participants nearly unanimously described negative experiences within religious institutions. Religious condemnation of same-sex sexual behavior was frequently discussed, including religious leaders preaching against same-sex behavior and congregations kicking out members who came out as gay or bisexual. In response to this rejection, participants described avoidance of religious institutions and/or concealment of their sexual orientation in religious contexts. Below, Okeyo (27, gay, Kisumu) describes the impact on his life of not being able to go to church.

*“So if you are a gay person and you don’t even go to church, you will feel left out. This thing again is a stress. You are like—I mean I’m not normal. People go to church. I cannot even go to church.…. I want to go to church with my partner and share this like any other person. …This is a challenge and it’s also bringing more stress within the gay community.”*

In addition to enacted discrimination and harassment, participants described experiencing stigma in religious contexts in the form of widespread religious misconceptions on the origins of same-sex sexual behavior. These misconceptions include the beliefs that same-sex behavior in Africa is a symptom of a curse/witchcraft or a ploy for the Devil to eradicate Africans with HIV. Below, Paul (38, gay, Nairobi) describes the power of religious leaders to influence widespread cultural attitudes towards gay and bisexual men.

*“…you know religious leaders…they’re [the] mouthpiece of the community. What they say is final. If they say today all gay men should be banned, unfortunately, the community will go about it. They’ll say, ‘Ok. Right. They should not be there.’…they believe we are sinners: we are not supposed to be in the society and we should repent- we have a problem. And we don’t. We don’t have a problem. This is who we are.”*

#### 3.2.2. Employment Institutions

Manifestations of stigma within employment institutions included discrimination and harassment from co-workers and supervisors, which presented difficulties in acquiring and maintaining steady work. Below, Ochieng (24, bisexual, Kisumu) describes the risk of being fired for being gay/bisexual.

*“…particular employers will not want to associate with you… If you are gay, if you are bi or something, they feel like … you are going to create that bad image…about this particular organization or company. So in cases when that is realized [about] you, [they] won’t communicate to you directly … but you find … that you just may be suspended, you will be expelled….”*

Participants noted that stigma in the workplace, especially for more feminine-presenting men, may emerge as being arbitrarily denied a position despite their qualifications for the job, being terminated from a position because of their sexuality, or experiencing hostility in the workplace. Additionally, participants noted that psychological distress stemming from discrimination inside or outside the workplace may hinder their job performance and consequently result in them leaving or being terminated from a position. Below, Mwangi (39, bisexual, Nairobi) describes the stress of being denied employment because of one’s sexual orientation.

*“…maybe you’re wanting to work somewhere. Then once they realize that you’re gay, they don’t give you the job. So that one would affect you mentally.”*

#### 3.2.3. Educational Institutions

Manifestations of stigma and violence within educational institutions included discrimination and harassment preventing gay and bisexual men from finishing their education or performing at the highest possible level. Participants discussed being kicked out of school because of their sexuality as well as being unable to continue their education because of bullying or a lack of financial support from family. The understanding that being outed could lead to punishment or expulsion from school created a need for constant concealment of one’s sexual orientation. Below, Ahmed (42, gay, Kisumu) describes the impact of being outed on one’s ability to complete secondary education.

*“…they are even thrown away from schools, they are being expelled from school because they are gay. And when you…ask for even a letter for a transfer, when you are looking for another school maybe you won’t be given…. So they will take time to even call for the teacher, [to see] what happened. And when they investigate and find that…you are gay, they won’t even admit you. So they end up…not even competing their secondary level education.”*

Participants also discussed how challenges outside of school settings can be barriers to education; for example, mental health challenges, substance use, or stigma and discrimination from outside sources can make it impossible for gay and bisexual men to focus on their studies and succeed academically. Below, Kamau (26, gay, Nairobi) describes the impact of outside stressors on one’s academic success.

*“… they find it’s [education] not a need for them because the- the situation they are going to face. Or probably the challenges on the mental issues they already face. So it becomes really hard to concentrate and read and say, ‘Man, let me concentrate on this book, eh.’ But it still- your mind is not there because mentally, you’re either depressed, stressed, or going through something.”*

#### 3.2.4. Healthcare Institutions

Manifestations of stigma and violence within healthcare institutions included discrimination, harassment, and rejection in hospitals, clinics, and other healthcare settings. Participants reported being denied treatment and rejected from healthcare settings once staff members learned of their sexual orientation. In particular, participants discussed the extreme difficulty of receiving care for anal STIs (particularly, anal warts) because such conditions reveal to providers that the individual has had anal sexual penetrations and is likely gay or bisexual. Below, Mike (30, gay, Kisumu) describes his experience being denied care.

*“…for example, there was a time when I was having anal warts…I went to the public hospital…the doctor asked me ‘What? What’s happened?’ And they asked me, ‘Were you raped?’ And I did not have [an] answer to answer them. So, they asked me, ‘Are you a gay? Because you talk like a gay. We cannot treat gays.’ And I was chased away.”*

This stigma and discrimination prompted participants to seek care at LGBTQ+ friendly clinics rather than hospitals for sexual health concerns, but also had severe mental and physical implications including the stress of concealment and the pain of untreated conditions. Below, Omondi (23, bisexual, Kisumu) describes the mental and physical issues stemming from avoidance of care.

*“…some of the rural parts actually don’t have free access to the, even they fear going to the general hospital because they feel like, ‘Oh, the doctor might do this and this’. Yeah. So they get that inner feeling, they feel depressed, and after all we just find them suffering from maybe an STI that could have been treated long ago, only because they fear or the doctor might out them…”*

## 4. Discussion

This study utilized a qualitative approach to examine interpersonal and institutional violence and stigma faced by Kenyan gay and bisexual men. Of the seven themes that emerged describing sources of violence and stigma, three described intimate interpersonal sources (family, friends, and romantic and/or sexual partners) and four described critical institutional sources (religious, employment, educational, and healthcare institutions). In addition, four sub-themes provided more extensive information regarding the various ways in which violence and stigma were reported within romantic and sexual relationships, including gay-baiting violence, blackmail, intimate partner violence, and commitment phobia.

These findings are of particular significance since all the identified sources of violence and stigma within both the interpersonal and institutional domains could be potential resilience resources for mitigating negative physical and mental health outcomes if they provided affirmation and support as opposed to violence and stigma. For example, in line with existing literature [27], some participants described the positive influence of possessing individual-level intrapersonal religious beliefs on their mental and physical health. However, participants also described significant stigma and violence within religious institutions, which negatively impacted their mental health and, in some cases, placed them in physical danger. These data reveal that Kenyan gay and bisexual men experience damaging and pervasive stigma and violence in the critical domains of their life that would typically provide support and strength, thus potentially placing them at risk for negative physical and mental health outcomes.

The current study findings are in line with what has been reported in other studies. Notably, the Human Rights Watch’s 2015 report The Issue is Violence, and the Kenya Human Rights Commission’s 2011 report, The Outlawed Amongst Us, both present qualitative data demonstrating the severity and pervasiveness of violence faced by Kenyan gay and bisexual men. While the primary goals of these reports were to identify patterns rather than sources of violence, both reports describe stigma, discrimination, abuse, and harassment from all seven sources identified in this analysis [1,2]. Of note, these reports also included descriptions of violence from state actors, such as police, which did not appear in this analysis. It is possible that this omission is due to the design of the interview guide, which specifically asked about positive and negative experiences with the sources identified here but not specifically about state actors.

These findings are also in line with research on the mental and sexual health of Kenyan gay and bisexual men. For example, qualitative data from Graham et al. [34] and quantitative data from Mbeda et al. [40] highlight the pervasiveness and negative impact of anti-LGBTQ+ stigma within healthcare institutions. However, both of these studies are focused on HIV prevention/treatment. While data from studies within an HIV-related context are valuable, they are limited in both their generalizability to the broader community of Kenyan gay and bisexual men and in their conceptualization of health as focusing only on HIV status.

Although this study did not seek to affirm or empirically validate the Minority Stress Model [19], the findings do lend additional qualitative support for the utility of this framework in understanding factors that contribute to mental and physical health challenges among gay and bisexual men in Kenya. Our focus on sources of stigma and violence in both interpersonal and institutional domains are considered distal stressors since they are external events and conditions. The narratives of our participants also revealed a range of proximal stressors or cognitive processes that can contribute to their distress. The predominant proximal stressors in the model are self-stigma, concealment, and expectations of rejection [19], all of which were discussed in various forms throughout these results. The desire to conceal one’s sexual orientation from family and friends, as well as within all four of the critical social institutions we explored, was perhaps the most predominant overarching phenomena across all themes and suggests a high level of proximal stress as gay and bisexual men attempt to keep their true identity concealed in so many settings and with the most important people in their lives. Participants’ fears and expectations of rejection from these important people and institutions could also be noted in their narratives, and at times led participants to avoid potentially supportive people and institutions out of fear of rejection. Although not as common in the narratives, suggestions of self-stigma were present as well, as some participants internalized the negativity they received from others.

### 4.1. Implications for Practice

These findings may be used to identify targets for sensitization campaigns to reduce the stigma and discrimination that Kenyan gay and bisexual men experience. By tailoring sensitization campaigns to the sources of stigma and violence identified here, these campaigns could more effectively promote acceptance and reduce the burden of stigma on the lives of community members. For example, education campaigns to dispel cultural and religious myths around same-sex sexuality could potentially be a tool for reducing downstream stigma, discrimination, and harassment. While the need to train healthcare providers in Kenya on the unique needs of gay and bisexual men has already been identified in the literature [41], these data point to similar opportunities for intervention from multiple interpersonal and institutional sources.

Findings from this study could also be used to inform resilience-promotion interventions based on the identification of interpersonal and institutional resources from which Kenyan gay and bisexual men are not receiving adequate support. For example, peer support is beneficial for well-being [33,42], but participants in this study described significant challenges to maintaining strong friendships (i.e., being cut off by friends after coming out). As such, an intervention designed to strengthen the social connections of Kenyan gay and bisexual men could buffer the impact of stigma from other sources, as described by the Minority Stress Model [19].

Perhaps the most significant implication of these findings is the importance of advocacy and policy change to destigmatize the identities of Kenyan gay and bisexual men and create systemic change to reduce the violence and stigma which this population faces. For example, socioeconomic stability has been recognized as a critically important resilience asset for Kenyan gay and bisexual men, and participants described criminalization as profoundly impacting their socioeconomic status [27]. Criminalization allowed gay and bisexual men to be arbitrarily denied or fired from a job because of their sexual orientation and allowed young gay and bisexual men to be expelled from schools and unable to complete their education. While this points to the urgency of creating programs to promote socioeconomic stability for Kenyan gay and bisexual men, it also demonstrates the importance of decriminalization of same-sex behavior to improve health outcomes at a larger level.

As described in these results, criminalization not only reflects socio-cultural anti-LGBTQ+ stigma, but also facilitates the proliferation of such stigma. For example, criminalization was described as normalizing violence against gay and bisexual men and made it extremely difficult or impossible to report abuses. This, in turn, increased vulnerability to various forms of violence, including blackmail and physical abuse, and negatively impacted physical and mental health. For example, fear of discrimination in healthcare institutions was described as resulting in delays in receiving care for urgent medical concerns. Similarly, the inability to live with one’s partner was described as necessitating secrecy in a relationship, reducing the perceived feasibility of long-term relationships, and making individuals vulnerable to blackmail. These findings point to the critical urgency for advocacy and political organizing to challenge the sections of the Kenyan Penal Code which criminalize consensual same-sex sexual behavior, as this criminalization permits routine human rights abuses, denies gay and bisexual men their constitutional rights to equal treatment, and has profound implications for health outcomes and associated vulnerabilities.

### 4.2. Future Research, Strengths, and Limitations

These data also can be used to inform future research. As this paper only examines violence and stigma at interpersonal and institutional levels, future studies could examine these issues at other socio-ecological levels. Data collection on how patterns of violence and stigma vary across the country may also be useful, as the data for this study were aggregated from all study sites for analysis. Future studies could focus on how experiences of violence and stigma vary across geographic location, rural/urban areas, religious affiliation, socioeconomic status, or other relevant factors. It is important to note that these data focus only on gay and bisexual men, which excludes other members of the Kenyan LGBTQ+ community, who may have vastly different experiences with stigma and violence. As such, research into sources of stigma faced by lesbian and bisexual women and by transgender and other gender minority individuals is urgently needed to better understand and address the needs of those communities, as they have been widely excluded from health promotion research and interventions [11,43]. Finally, since these data were collected prior to the COVID-19 pandemic, future research could examine the impact of COVID-19 on the lives of gay and bisexual men in Kenya, including on experiences of violence and stigma. Globally, the COVID-19 pandemic disproportionately impacted communities that are highly marginalized, and these results highlight some of the preexisting vulnerabilities of this community [44,45].

One limitation of the current paper is that data were only collected in two of Kenya’s largest cities, so the experiences of men living in urban areas and rural regions outside of these cities were not captured. A second limitation is that, since participants were recruited from community-based organizations, gay and bisexual men who are highly closeted and do not interact with such organizations may not have been reached. These men may have different experiences with violence and stigma based on varying levels of conformity with traditionally masculine, heteronormative gender roles and gender presentations. The predominant strength of this paper is that data were collected as part of a community-centered study that was developed in response to Kenyan gay and bisexual men requesting assistance with data collection that was focused on the lived experiences of community members and not focused on HIV. The semi-structured qualitative interview guide was developed in a collaborative manner by the original research team, most of whom are Kenyan gay and bisexual men, and all interviews were conducted in safe places by trusted community leaders. These factors enhanced the cultural relevance of the interview guide and provided a safe environment where participants could describe their experiences in their own words. Another strength of the current paper lies in the phenomenological qualitative approach to data analysis which allowed us to further center the voices of community members.

## 5. Conclusions

Gay and bisexual men in Kenya face stigma and violence within intimate interpersonal relationships as well as within critical social institutions which are intended to support health. As indicated by the Minority Stress Model, stigma and violence have severe negative implications for the well-being of Kenyan gay and bisexual men, including for their physical, mental, and sexual health. The qualitative data presented here not only identify interpersonal and institutional sources of stigma and violence faced by Kenyan gay and bisexual men but also describe the ways in which such stigma and violence manifest and impact the lives of community members. The use of open-ended interviews and a qualitative, phenomenological approach allowed us to center the voices of community members. These data may be used to identify targets for sensitization campaigns, inform health promotion interventions, and advocate for policy change to destigmatize and protect gay and bisexual men in Kenya.

## Figures and Tables

**Table 1:** Participant Demographic Data.

	Participants (*n* = 60) *	
Age	Mean Age: 29.3 Years	Range: 20–46 Years
Highest Education Level	Primary school	3 (5.1%)
	Secondary school	15 (25.4%)
	Certificate	7 (11.9%)
	Diploma	24 (40.7%)
	Bachelor’s degree	8 (13.6%)
	Master’s degree	1 (1.7%)
	Currently attending school	1 (1.7%)
Employment Status	Part-Time	23 (39.0%)
	Full-Time	25 (42.3%)
	Casual Laborer	2 (3.4%)
	Sex Worker	2 (3.4%)
	Not working but in school	1 (1.7%)
	Not working and not in school	2 (3.4%)
	Other	4 (6.8%)
Current Gender	Male/Man	60 (100%)
Sex Assigned at Birth	Male	60 (100%)
Sexual Orientation	Gay/Homosexual	45 (76.3%)
	Bisexual	14 (23.7%)
Religion	Catholic	22 (37.3%)
	Anglican	14 (23.7%)
	Other	7 (11.9%)
	Seventh Day Adventist	6 (10.2%)
	Muslim	5 (8.5%)
	Indigenous	3 (5.1%)
	None	2 (3.4%)
County	Kisumu	32 (53.3%)
	Nairobi	28 (46.7%)
Ethnic Group	Luo	32 (55.2%)
	Kikuyu	8 (13.8%)
	Luhya	7 (12.1%)
	Kamba	3 (5.2%)
	Other/Multiple	3 (5.2%)
	Kish	2 (3.4%)
	Baganda	1 (1.7%)
	Swahili	1 (1.7%)
	Tutsi	1 (1.7%)

* Note some sections do not add up to 60 because of a small number of missing responses.

**Table 2:** Code tree.

Interpersonal Level	Institutional Level
(1) Family	(1) Religious Institutions
(2) Friends	(2) Employment Institutions
(3) Romantic and/or Sexual Partners	(3) Educational Institutions
(3a) Gay-Baiting Violence (3b) Blackmail (3c) Intimate Partner Violence (3d) Commitment Phobia	(4) Healthcare Institutions

## Data Availability

The data presented in this study may be available on request from the corresponding author. The data are not publicly available due to the sensitive and potentially identifying nature of the qualitative data collected.
